# First record of a mermithid worm (Nematoda, Mermithidae) parasitizing a third instar nymph of *Triatoma sordida* (Stål, 1859) (Hemiptera, Reduviidae, Triatominae) from Mato Grosso, Brazil

**DOI:** 10.3897/zookeys.980.55865

**Published:** 2020-10-28

**Authors:** Mirian Francisca Martins, Sinara Cristina de Moraes, Simone Chinicz Cohen, Melissa Querido Cárdenas, Cleber Galvão

**Affiliations:** 1 Departamento de Vigilância em Saúde Ambiental, Secretaria de Estado de Saúde de Mato Grosso – SESMT. Amaro Leite, 474, Barra do Garças, MT, 78600–000, Brazil Secretaria de Estado de Saúde de Mato Grosso Barra do Garças Brazil; 2 Programa de Pós-Graduação em Biodiversidade e Saúde, Instituto Oswaldo Cruz, FIOCRUZ, Av. Brasil 4365, Rio de Janeiro, RJ, 21040–360, Brazil Instituto Oswaldo Cruz Rio de Janeiro Brazil; 3 Laboratório de Helmintos Parasitos de Peixes, Instituto Oswaldo Cruz, FIOCRUZ, Av. Brasil 4365, Pavilhão Cardoso Fontes, sala 45, Rio de Janeiro, RJ, 21040–360, Brazil Secretaria de Estado de Saúde de Mato Grosso Barra do Garças Brazil; 4 Laboratório Nacional e Internacional de Referência em Taxonomia de Triatomíneos, Instituto Oswaldo Cruz, FIOCRUZ, Av. Brasil 4365, Pavilhão Rocha Lima, sala 505, Rio de Janeiro, RJ, 21040–360, Brazil Instituto Oswaldo Cruz Rio de Janeiro Brazil

**Keywords:** Mato Grosso, Brazil, Mermithidae, Nematoda, new host record, *Triatoma sordida*, Triatominae, parasite

## Abstract

A juvenile specimen of a mermithid (Nematoda) was found parasitizing a third instar nymph of *Triatoma sordida* from Mato Grosso, Brazil. This is the first record of mermithid parasitism in a triatomine species. The Mermithidae represents a family of nematodes that are specialized insect parasites. Entomonematodes are one of the highly influential agents regulating the population dynamics of insects. This report introduces the opportunity to think about mermithids as a possible candidate for use as triatomine biological control.

## Introduction

The insects of the subfamily Triatominae (Hemiptera, Reduviidae) are true bugs specialized in blood-sucking. All species are potential vectors of *Trypanosoma cruzi* (Chagas, 1909) (Trypanosomatida, Trypanosomatidae), the causative agent of Chagas disease in the Americas, where the disease remains an important public health problem. Although a few species of triatomines are also found in Asia and Oceania, in these regions the vector-borne transmission of *T. cruzi* does not occur as the parasite is absent. Galvão and Justi (2015) summarized the information available about ecology, niches, and associations with humans, and *T. cruzi* infection of all triatomine species.

*Triatoma sordida* (Stål, 1859) is a species endemic to Argentina, Bolivia, Brazil, Paraguay, and Uruguay (Galvão 2014). In Brazil, *T. sordida* is the most commonly collected triatomine species, predominantly in peridomiciliar environments (Obara et al. 2011; Rossi et al. 2015). *Triatoma sordida* presented the highest infection rate for *T. cruzi* in the state of Bahia, including the report of infected colonies in intradomiciary environments (Ribeiro-Jr. et al. 2019). According to Minuzzi-Souza et al. (2017), *T. sordida* plays a key role in maintaining the risk of transmission of *T. cruzi* to humans in the state of Góias and the Distrito Federal in Brazil. In the state of Bahia, Brazil, this species was associated with oral transmission of Chagas disease to humans (Dias et al. 2008).

Entomonematodes are one of the highly influential agents regulating the population dynamics of insect pests through association with their hosts in relationships ranging from fortuitous to parasitic. Many investigators have recognized these parasites as potential biological control agents (Poulin 2011). Results showed that entomonematodes are a safe and effective environmental alternative for controlling pests in crops of economic importance (Shapiro-Ilan et al. 2010; Duncan et al. 2013; Gumus et al. 2015), because of their capacity to retard development, induce female sterility, and cause death on host emergence (Kaiser 1991).

The mermithids represent a family of nematodes with more than fifty genera, specialized parasites of invertebrates, especially insects, parasitizing at least fifteen different orders (Nickle 1972; Poinar-Jr. 2015). The life cycle of mermithids includes five stages, as described in Poinar-Jr. (1983): egg, second stage juvenile (preparasitic infective juvenile), parasitic third stage juvenile, mature third stage postparasitic juvenile, two molts into adult (Kosulic and Masova 2019). The eggs of nematodes of terrestrial insects, containing juvenile forms, are deposited under leaves for ingestion as food. After ingestion, the eggs break, releasing juveniles that migrate to the insect hemolymph (Dolinski 2006). Another route involves the migration of the preparasitic larvae to the soil surface and climb grass or other vegetation to reach their hosts, when hatching occurs. They penetrate the body wall of recently hatched nymphs, entering in the body cavity. Within the host, the parasitic complete their growth and then emerge by forcing their way through the body wall to enter the soil (Christie 1936). Nematodes kill the host with their emergence to the soil where they molt into the adult stage to complete the cycle (Poinar-Jr. 1979). Emergence from its host by killing it, places them as parasitoids (Rusconi et al. 2017).

These parasitoids have parenteral intake of nutrition from the host tissues and hemolymph, which may strongly influence the physiological condition of the host, from the first instars of parasite development (Nikdel et al. 2011), promoting severe competition for nutrients, resulting in atrophy of the thorax and abdomen, organ involvement, and changes in patterns of development of social insects and the general behavior of insects (Dolinski 2006). Parasitism by a mermithid is fatal to the host (Nickle 1972; Poinar-Jr. 1983; Nikdel et al. 2011).

Postparasitic juvenile and adult mermithids are most frequently collected. In this postparasitic free-living stage, the parasite does not feed anymore and only needs a suitable habitat to mature (Kosulic and Masova 2019).

Information about Mermithidae nematodes is scarce. In South America, there are a few studies about terrestrial mermithids in grasshoppers (Miralles and Camino 1983; Camino and Stock 1989; Camino and Lange 1997; Rusconi et al. 2017) and ants (Jouvenaz and Wojcik 1990; Poinar-Jr. et al. 2007) from Argentina. In Brazil, Rodrigues et al. (2005) reported an unidentified Mermithidae larva emerging from spiders from Brazil and Peru.

During entomological research for triatomine in the municipality of Araguaiana, Mato Grosso, Brazil, a single specimen of a mermithid nematode was collected from *T. sordida*. The purpose of the present paper is to report the first finding of a juvenile stage of a mermithid nematode parasitizing Triatominae from Brazil. To date, there are no records of endoparasitism by nematodes in triatomines in the world. A list of records of mermithids from hemipteran hosts is presented in Table 1.

**Table 1.** Worldwide mermithid host records for Hemiptera.

Host species	Genus	Locality	Reference
*Acrosternum hilare* (Say, 1832) *Euschistus servus* (Say, 1832)	* Hexamermis *	United States	Kamminga et al. (2012)
*Aelia acuminata* (Linnaeus, 1758)	Undetermined	Uzbekistan	Sultanov et al. (1990)
*Aelia rostrata* Boheman, 1852	* Hexamermis *	Turkey	Tarla et al. (2012)
	* Mermis *		Memişoğlu and Özer (1994)
*Chinavia hilaris* (Say, 1831)	* Hexamermis *	United States	Kamminga et al. (2012)
	* Agamermis *		Stubbins et al. (2016)
*Coptosoma mucronatum* Rubostov, 1977	* Pentatomermis *	Slovakia	Rubtsov (1977)
*Euschistus servus* (Say, 1832)	Undetermined	United States	Esquivel (2011)
	* Agamermis *		Stubbins et al. (2016)
*Euschistus* sp.	* Agamermis *	United States	Stubbins et al. (2016)
*Eurygaster integriceps* Puton, 1881	* Mermis *	Turkey	Dikyar (1981)
	* Hexamermis *		Memişoğlu and Özer (1994)
	* Hexamermis *	Turkey	Tarla et al. (2011)
*Eurygaster maura* (Linnaeus, 1758)	* Hexamermis *	Turkey	Tarla et al. (2015)
*Elasmostethus interstinctus* (Linnaeus, 1758)	* Pentatomermis *	Russia	Rubtsov (1969)
*Halys dentatus* (Fabricius, 1775)	* Hexamermis *	India	Yadav and Dhiman (2004)
*Megacopta cribraria* (Fabricius, 1778)	* Agamermis *	United States	Stubbins et al. (2016)
*Nezara viridula* (Linnaeus, 1758)	Undetermined	United States	Fuxa et al. (2000)
	* Pentatomermis *	India	Rubtsov (1977) Bhatnagar et al. (1985)
*Euschistus* spp.	* Agamermis *	United States	Stubbins et al. (2016)
*Platynopus* sp.	* Hexamermis *	India	Gokulpure (1970)
*Piezodorus guildinii* (Westwood, 1837)	Undetermined	United States	Kamminga et al. (2012)
	*Hexamermis* or *MermisHexamermis*	Uruguay United States	Riberiro and Castiglioni (2008) Kamminga et al. (2012)
*Rhaphigaster nebulosa* (Poda, 1761)	* Hexamermis *	Italy	Manachini and Landi (2003)
*Sogatella furezfera* (Horvath, 1899)	* Agamermis *	Asia	Choo and Kaya (1993)

## Materials and methods

The municipality of Araguaiana is located in the northeast Mesoregion of Mato Grosso state, Brazil (15°43'47"S, 51°49'26"W), 270 m high, with 3,197 inhabitants spread over 6,429,386 km^2^. The predominant biome is the Cerrado (IBGE 2012) and the main economic activity is livestock production (IMEA 2017).

The climate is characterized by two main seasons (dry winter and rainy summer), corresponding to type Aw according to the Köppen classification (Köppen 1948). The average annual humidity is 60% and the average annual temperature 24 °C, with a maximum of 40 °C and lowest minimum of 4 °C.

During the years 2017 to 2019, 28 rural localities of Araguaiana, Mato Grosso, Brazil, were monitored as part of entomological research for triatomine bugs. Insects were manually collected by the method of active search with the support of health surveillance agents from the “Secretaria Municipal de Saúde” of Araguaiana, inside the domicile environment from domiciliary units (DUs) with evidence of the presence of triatomines and/or reports of the presence of the bug by the resident and around artificial ecotopes in domicile environments. For the DUs with the presence of a triatomine the geographical coordinates were taken with a GPS Garmin.

In the entomological laboratory of the “Escritório Regional de Saúde de Barra do Garças” from the “Secretaria de Estado de Saúde de Mato Grosso” (ERSBG/SESMT), the insects were counted, separated according to developmental stage (nymphs, adults) and posterior classification of the evolutionary stage of the nymphs and sex of the adults. These latter were taxonomically identified using the taxonomic keys of Lent and Wygodzinsky (1979) and Galvão (2014).

For investigation of the natural infection of *T. cruzi* in these triatomine specimens, dissection of the last portion of the abdominal segment was performed, slowly removing the entire intestine in the direction of a microscope slide, with the aid of tweezers.

For the taxonomic identification, the specimen of nematode worm was placed in a 2 mm tube containing 70% ethanol and sent to the “Laboratório de Helmintos Parasitos de Peixes” of the Oswaldo Cruz Institute, FIOCRUZ. The nematode was clarified in phenol and mounted on temporary slides. Measurements were taken directly using an ocular micrometer and are given in millimeters. Light microscope pictures were taken using a Zeiss Axioscope 2 microscope equipped with a camera lucida and a Sony MPEG Movie EX DSC-S75 digital camera. The nematode was identified according to available literature (Nickle 1972; Choo and Kaya 1990; Kaiser 1991; Stubbins et al. 2016).

## Results

**Figure 1.**
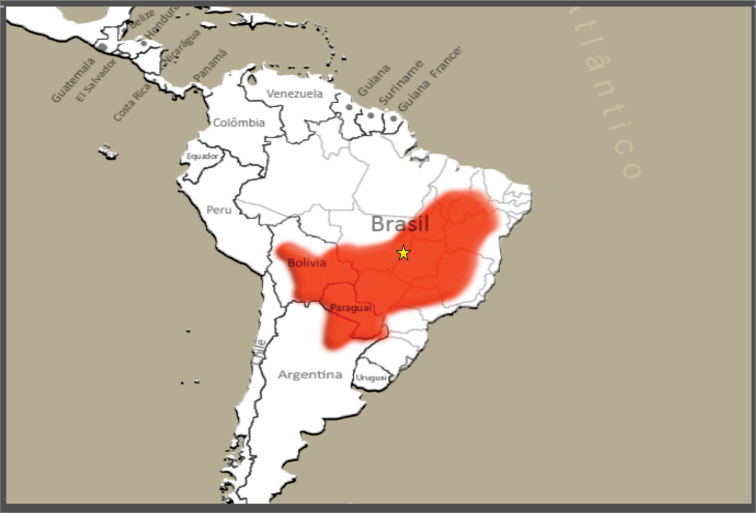
Map of the distribution of *Triatoma sordida*, showing the studied locality “Fazenda Lago Azul” in Araguaiana, Mato Grosso, Brazil (15°43'47"S, 51°49'26"W).

During entomological research for triatomines in the municipality of Araguaiana, Mato Grosso, Brazil, 1,488 specimens of *T. sordida* were found, with 220 being caught in the locality “Fazenda Lago Azul”, Mato Grosso, Brazil (Fig. 1).

**Figure 2.**
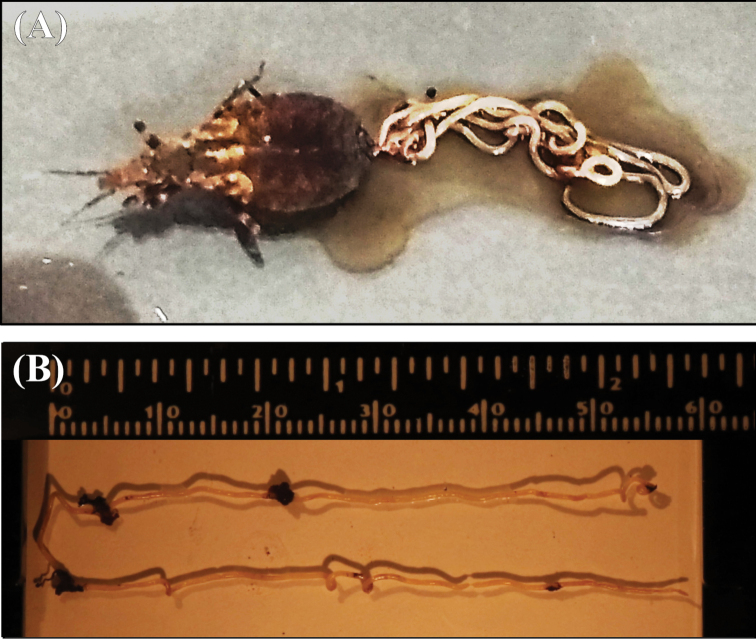
Mermithid: **A** specimen emerging from the posterior end of the third stage nymph of *Triatoma sordida* (Stål, 1859) collected in a chicken coop in Araguaiana, Mato Grosso, Brazil in December 2018 **B** specimen on microscope slide.

During the standard procedure for the extraction of intestinal content of a nymph of a third stage *T. sordida* (15 mm deep), a long and slim parasite was observed emerging from the triatomine (Fig. 2).

**Figure 3.**
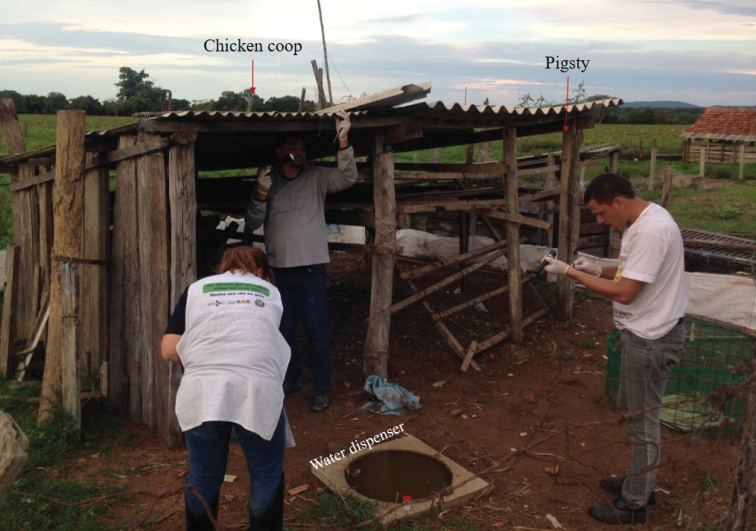
Chicken coop on “Fazenda Lago Azul”, Araguaiana, Mato Grosso, Brazil where the nymph of *Triatoma sordida* was found.

The parasitized specimen of *T. sordida* was collected in December 2018, in a chicken coop on “Fazenda Lago Azul”, Araguaiana, Mato Grosso, Brazil (15°33'43.9"S, 051°47'26.2"W, 294 m high). The triatomine nymph was found between wooden plates. One animal water dispenser was observed near the chicken coop (Fig. 3).

**Figure 4.**
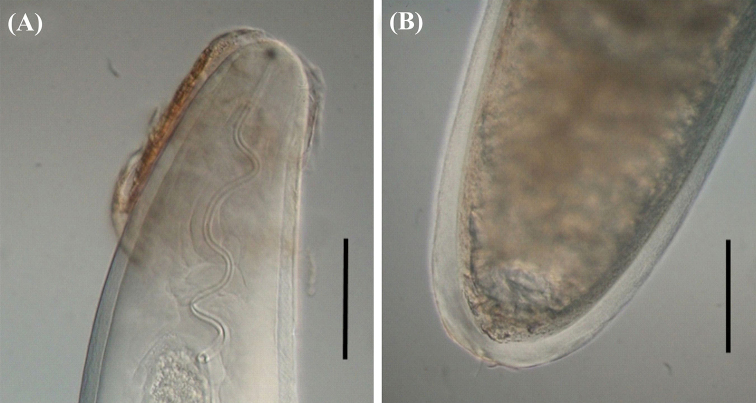
Mermithid nematode of *Triatoma sordida* observed by differential interference contrast (DIC). **A** Anterior portion **B** posterior portion showing the tail end ring. Scale bars: 60 mm (**A**); 150 mm (**B**).

Examination of parasitic juveniles under the microscope revealed well-developed stichosomes, a diagnostic characteristic of the family Mermithidae. The absence of tail appendage and presence of a tail end ring provided robust evidence for identification of the genus *Agamermis*. The specimen was white in color and slightly transparent at the tapered rounded ends (Fig. 4). The mermithid was extremely long in respect to its triatomine host, 193 mm in length and a maximum of 0.45 mm wide.

## Discussion

Although triatomines are obligatorily hematophagous in all phases of their development, feeding across a broad range of mammals and other vertebrate species, there are some species able to feed on invertebrates by kleptohematophagy, hemolymphagy, and coprophagy (Ruas-Neto et al. 2001; Sandoval et al. 2000, 2004, 2010; Otálora-Luna et al. 2015).

The chicken coop where the parasitized *T. sordida* nymph was collected showed the higher density of triatomines in artificial ecotopes in our entomological survey. It is noteworthy that December is a rainy season for this region, with record high rainfall and humidity for 2018. A high population density of triatomines can lead to increased local competition for food sources, leading insects to intense displacement in search of blood. Because they do not have wings, triatomine nymphs transit through the soil and an infective juvenile penetration from hatched eggs in the environment is supported (Christie 1936; Alves and Lopes 2008; Stubbins et al. 2016). Thus, it is likely that the juvenile form of this nematode present in the soil has penetrated in the nymph, explaining the presence of this mermithid parasitizing the *T. sordida*.

This study provides the first report worldwide of a mermithid nematode infecting the immature stages of the vector hemipteran, *T. sordida*. Terrestrial mermithids are a large group of obligate entomoparasitic nematodes that are considered important regulators for some insect populations, including hemipteran pests (Kaburaki and Imamura 1932; Choo and Kaya 1990), because of their capacity to retard development, induce female sterility, and cause death on host emergence (Kaiser 1991; Stubbins et al. 2016). The majority of mermithid species constitute a significant regulatory influence on the population dynamics of plague insects (Rusconi et al. 2017).

Females can migrate from the soil onto the vegetation and lay eggs during periods of high moisture. These eggs are later consumed by the insects along with the vegetal material and hatch in the gut; the juveniles subsequently pass through the gut wall into the hemocoel and considerably increase in size inside the host. Nematodes kill the host with their emergence to the soil where they molt into the adult stage to complete the cycle (Poinar-Jr. 1979; Rusconi et al. 2017).

There is robust evidence that the nematode Mermithidae found parasitizing *T. sordida* belongs to the genus *Agamermis* Cobb, Steiner and Christie 1923. For accurate genus and species identification adult samples are required. The determination of mermithid species is difficult. One reason for this is that often only larval forms are obtainable, and another is that mermithids do not possess obvious morphological characteristics (Cobb et al. 1923). It is estimated that only one fifth of nematode species have so far been described (Storer and Usinger 1991).

*Agamermis* spp. have been reported infecting many insects species all over the world, including Pentatomidae and Plastaspidae (Hemiptera), in the brown plant hopper (BPH), *Nilaparvata lugens* (Stål), and the white backed plant hopper (WBPH), *Sogatella furcifera* (Horvath), which are considered serious pests of rice Acrididae (Orthoptera), as well as in crustaceans in the Isopoda*Armadillidium vulgare* (Cobb et al. 1923; Kaburaki and Imamura 1932; Christie 1936; Weaver and King 1954; Rubtsov 1969; Choo and Kaya 1990; Baker and Poinar-Jr. 1995; Choo et al. 1995; Igbinosa 1988; Zimmerman 2010; Stubbins et al. 2016; Rusconi et al. 2017).

The Mermithidae family nematodes have been studied as a biological control mechanism with promising results (Dhiman and Yadav 2004; Stubbins et al. 2016). The growing concern about the negative environmental effects of controlling vector insects makes the discovery of alternative control agents essential.

This report introduces the opportunity of considering Mermithid parasites as possible candidates for use as biological control against Triatomines. The capacity of these parasites as regulators of the population, a mechanism essential to control Chagas disease should be investigated. Studies on nematode parasites of other hemipteran species showed that these parasites could demonstrate potential for population suppression.
